# Supplementary material for: Characterization of RNA Helicase Genes in Ustilago maydis Reveals Links to Stress Response and Teliospore Dormancy
Source: Int J Mol Sci. 2025 Mar 8;26(6):2432. doi: 10.3390/ijms26062432 (PMC11941951; doi:10.3390/ijms26062432)
Supplement: Supplementary file 1 [file ijms-26-02432-s001.zip › ijms-3481635-supplementary figures.pdf]

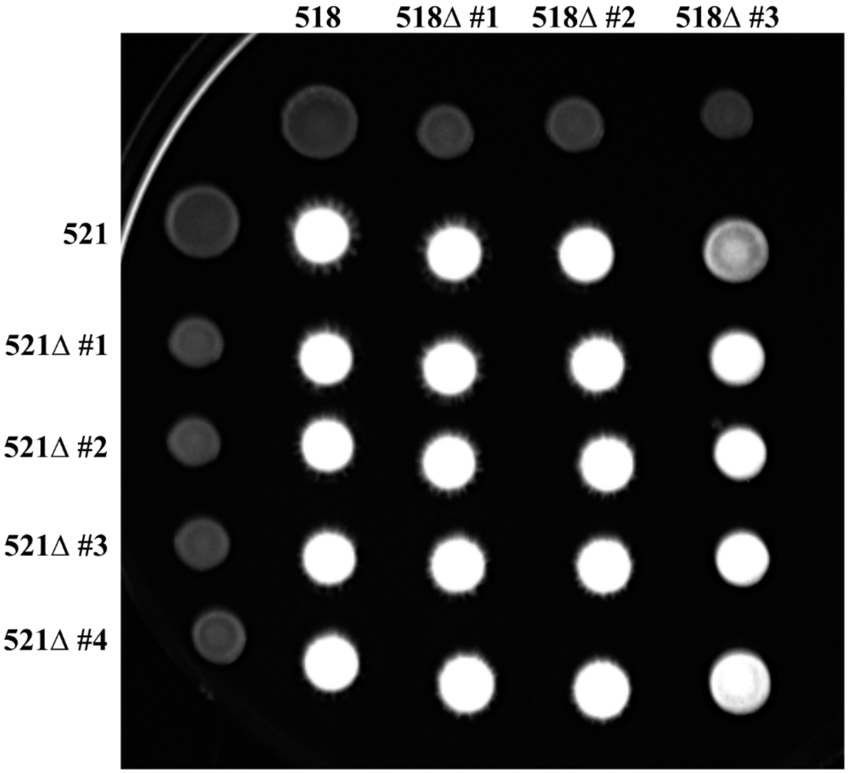

Figure S1. Forced dikaryon formation of *udbp3* deletion mutants. *Ustilago maydis* strains were cultured overnight in YEPS medium and normalized to an OD_{600} of 1.0. Equal volumes of compatible strains were premixed and spotted on solid PDA containing 1.0% activated charcoal. Plates were incubated at room temperature and filamentous growth was monitored for 3 days. Three technical replicates of the mating assay were performed, and the representative data is shown. The label abbreviation Δ indicates the Δ*udbp3* deletion mutant.

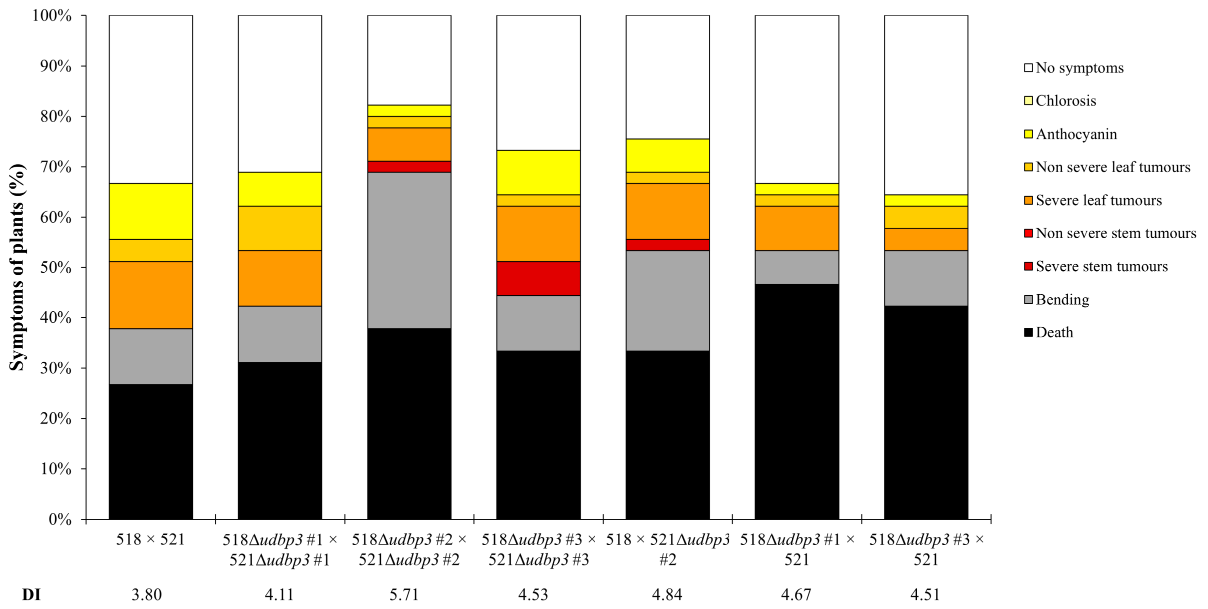

Figure S2. *udbp3* mutant pathogenesis assay in maize. Evaluation of virulence of *Ustilago maydis* 518 × 521 (wildtype), Δ*udbp3* × Δ*udbp3* (deletion), and Δ*udbp3* × wt (reciprocal) infections in *Zea mays*. Bars represent the percentage of plants displaying symptoms at 14 days post inoculation. Colours indicate the scored symptom ranging from mild (chlorosis) to severe symptoms (large stem tumours and plant death) as indicated in the legend. A total of 45 plants per cross were infected and the disease index (DI) is the calculated mean of the disease symptoms. Statistical differences were determined using Kruskal-Wallis test coupled with a Dunn multiple comparison ($p \leq 0.05$) and resulted in no significant difference between wildtype infections to deletion or reciprocal cross infections.

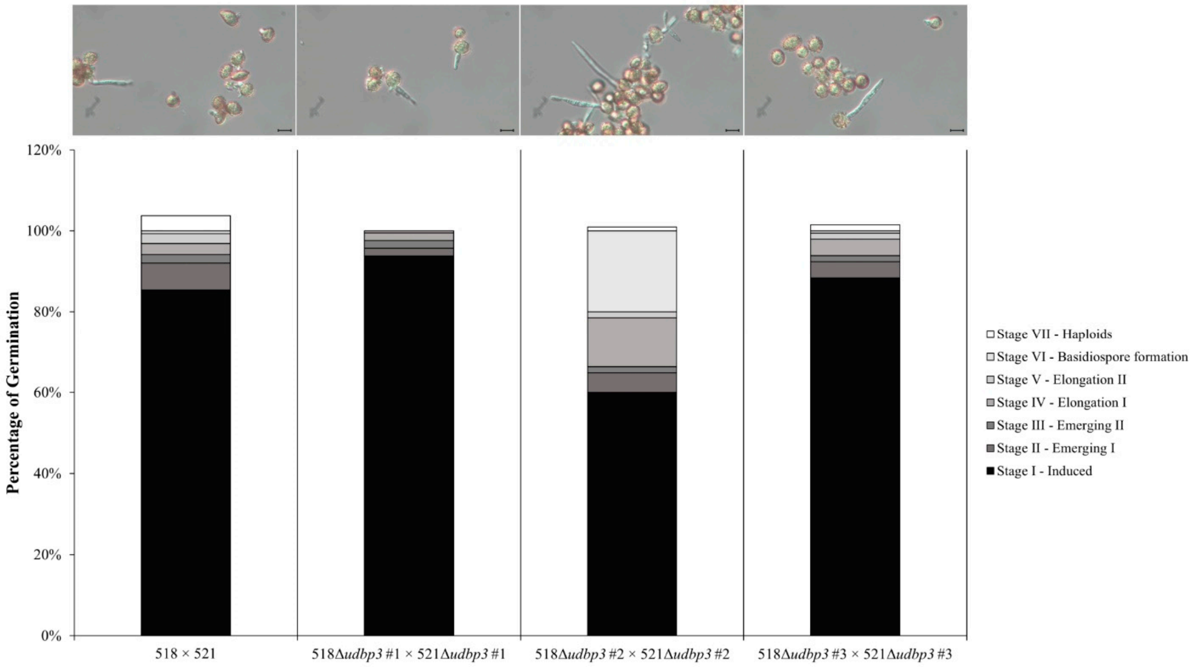

Figure S3. Teliospore germination test of *udbp3* deletion mutants. Teliospores were induced to germinate in YEPS Gold medium and incubated overnight. Germinating teliospores were harvested at 16 h post induction of germination for wildtype (518 × 521) and deletion (518Δ*udbp3* × 521Δ*udbp3*) crosses. Microscopic images of each sample were taken at 400× magnification and are displayed along the top. The percentage of germination was determined using a hemocytometer and stages of germination were identified based on the criteria in Seto*, et al.* [23]. Scale bar indicates 10 µm.

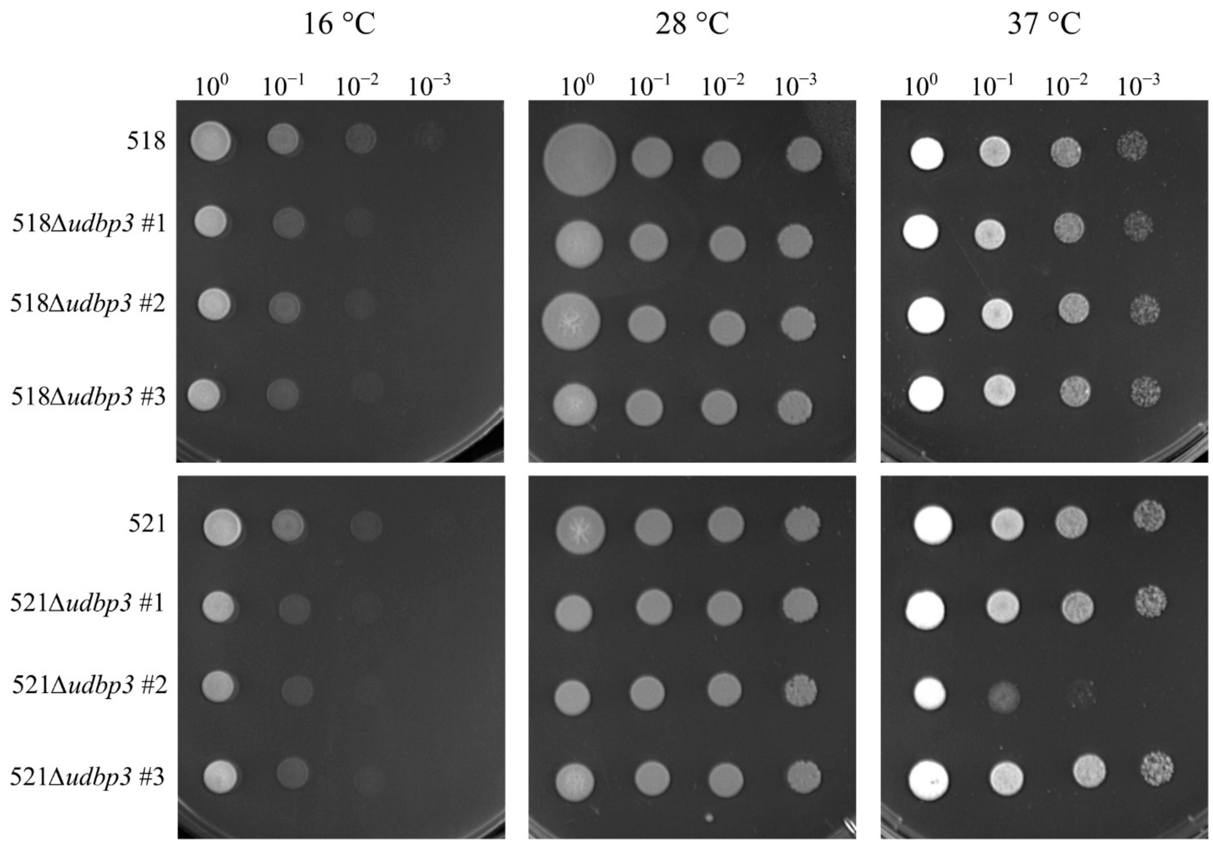

Figure S4. Growth of Δ*udbp3* at different temperatures. All strains were cultured overnight in YEPS Gold medium and normalized to an OD_{600} = 1.0. A 10-fold serial dilution series was created and spotted on MM medium containing 1.0% w/v D-glucose. Plates were incubated at either 16 °C, 28 °C, or 37 °C and monitored for 3 days. Photos were taken on the third day and the representative of three technical replicates of the spotting assay is shown.

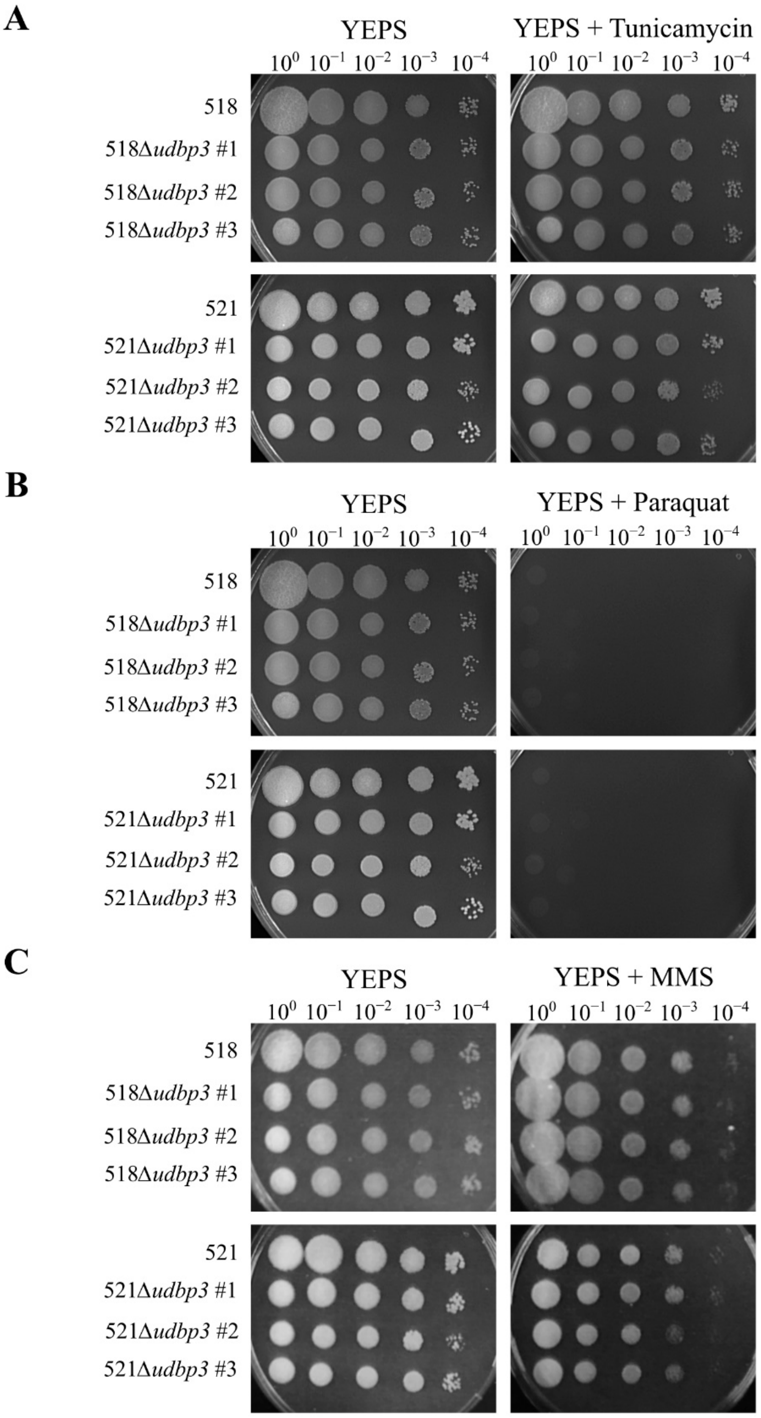

Figure S5. *udbp3* deletion mutants tolerance to various stressors. All *Ustilago maydis* strains were cultured overnight in YEPS Gold medium, washed, and normalized to an OD_{600} = 1.00. A 10-fold serial dilution series was created and spotted on YEPS and YEPS containing (A) 3 µg/mL tunicamycin, (B) 5 mM paraquat, and (C) 0.01% methyl methanesulphonate (MMS). All plates were incubated at 28 °C and monitored for 3 days. Photos were taken on the third day and the representative of three technical replicates of each spotting assay is shown.

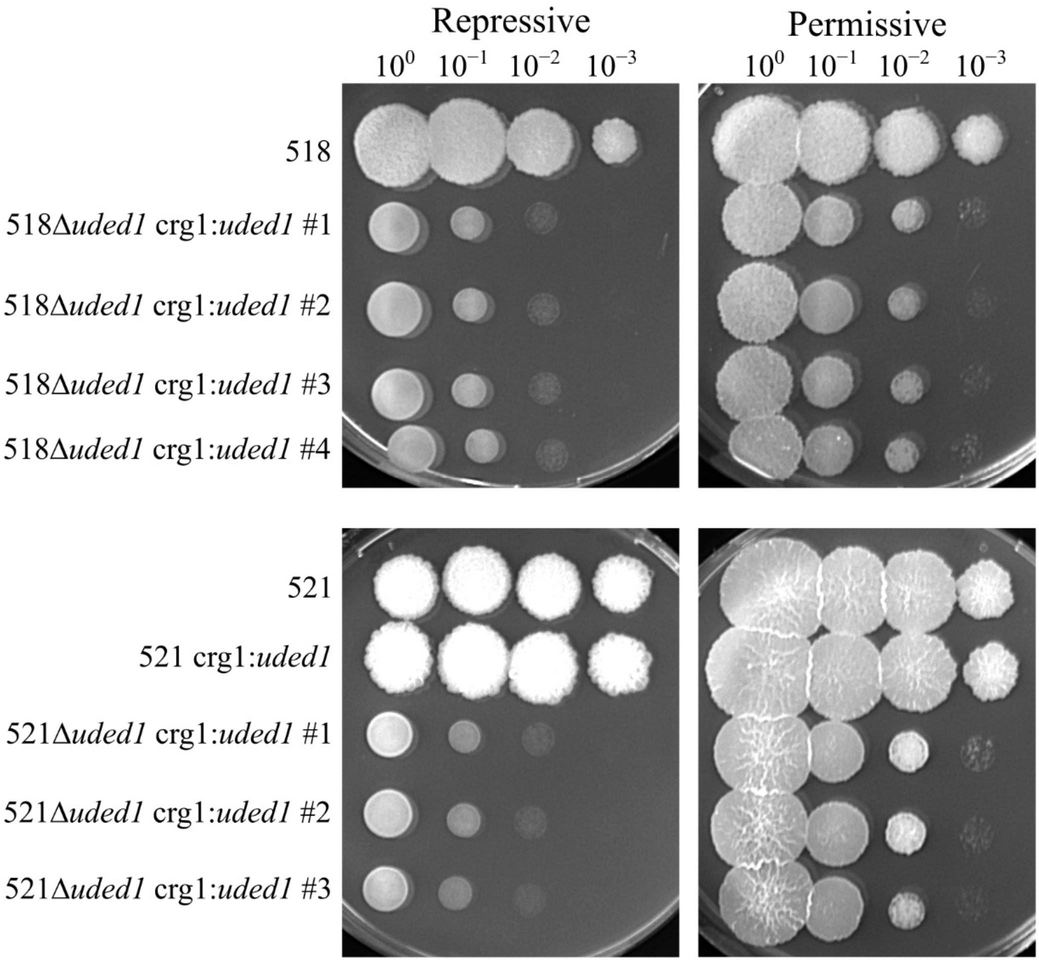

Figure S6. Growth of the *uded1* deletion strains in repressive and permissive growth conditions. A 10-fold serial dilution series was spotted on YEPS (repressive) and YEPA (permissive) plates. Plates were incubated at 28 °C and growth was monitored for 3 days. The data shown after 3 days of growth and is representative of three technical replicates of the spotting assay. The lighting of each photograph was adjusted using Inkscape v1.3.2.

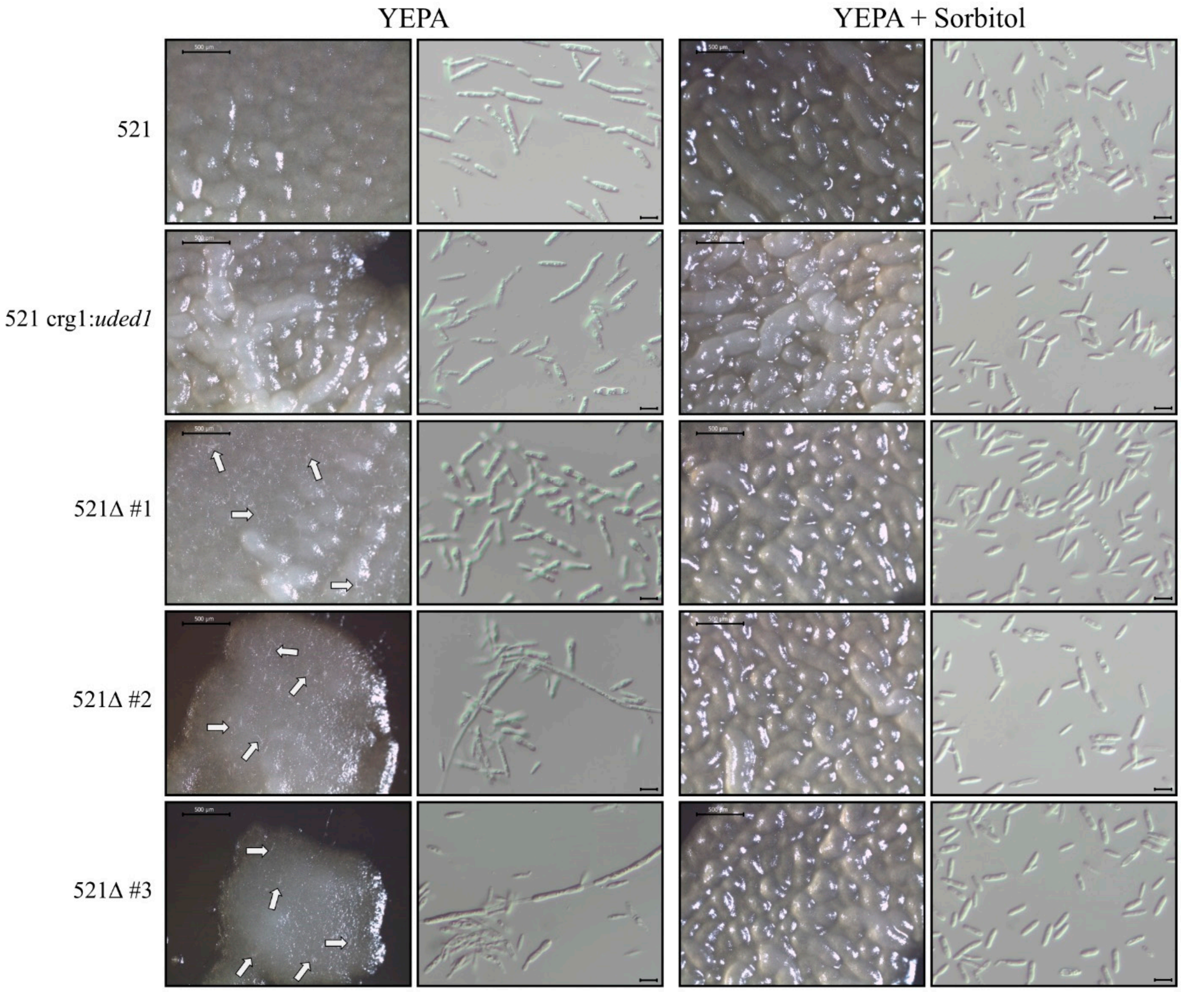

Figure S7. The effects of sorbitol addition to solid medium on the growth of *uded1* mutants. Single colonies of each *U. maydis* strain were streaked onto YEPA and YEPA containing 1 M sorbitol plates and incubated at 28 °C for 3 days. Microscopic images were taken of the growth on the plate (40×) and of the cells resuspended in sterile dH_2O (400× magnification). Scale bar indicates 500 µm (plate micrographs) and 10 µm (cell micrographs). Data shown is representative of three technical replicates of the growth assay. Arrows indicate mycelial growth. Label abbreviations: Δ#1–3 delineates the 521Δ*uded1* crg1:*uded1* biological replicates

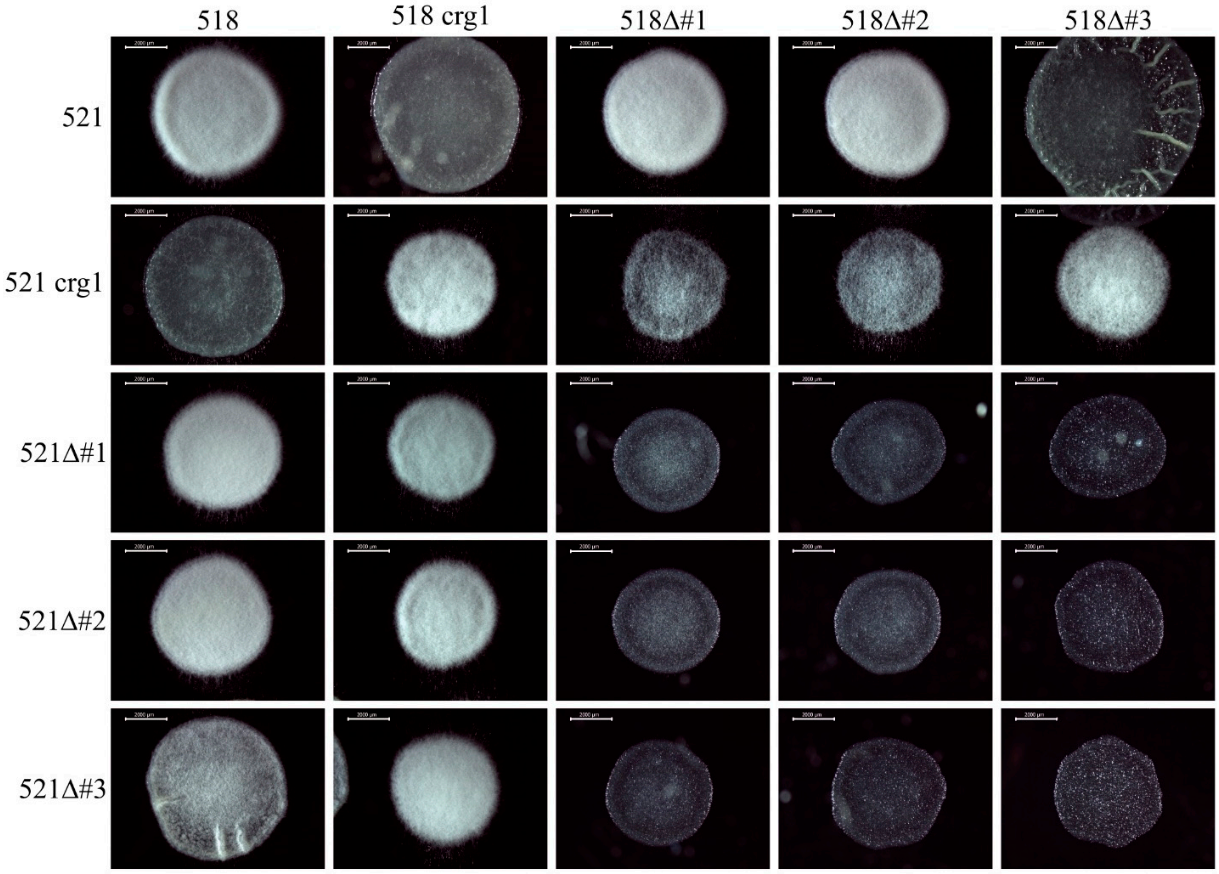

Figure S8. Mating assay of *uded1* mutants. All *Ustilago maydis* strains were grown overnight in DCM containing 1.0% w/v L-arabinose, washed, normalized to an OD_{600} of 1.0, and equal volumes of compatible strains were premixed. Premixed cultures were spotted on PDA containing 1.0% activated charcoal and incubated at room temperature for 3 days. Microscopic images of each plate were taken with a stereoscopic microscope. Scale bar = 2 mm and the representative data of three technical replicates of the mating assay is shown. The label abbreviations are: crg1 indicates the crg1:*uded1* mutants and Δ #1–3 indicates the Δ*uded1* crg1:*uded1* mutants.

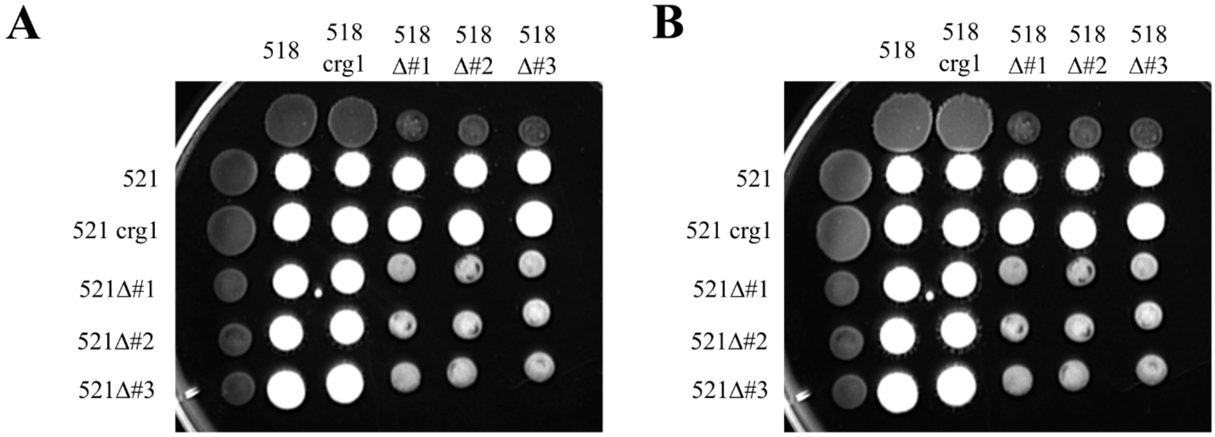

Figure S9. Mating assay of *uded1* mutants comparing three days versus five days. All *Ustilago maydis* strains were cultured overnight in DCM containing 1.0% w/v L-arabinose and 1 M sorbitol, washed, normalized to an OD_{600} of 1.0, and equal volumes of compatible strains were premixed. The premixed cultures were spotted on PDA containing 1.0% activated charcoal and incubated for (A) 3 days and (B) 5 days at room temperature. The representative data from three technical replicates of the mating assay is shown. The label abbreviations are: crg1 indicates the crg1:*uded1* mutants and Δ #1–3 indicates the Δ*uded1* crg1:*uded1* mutants.

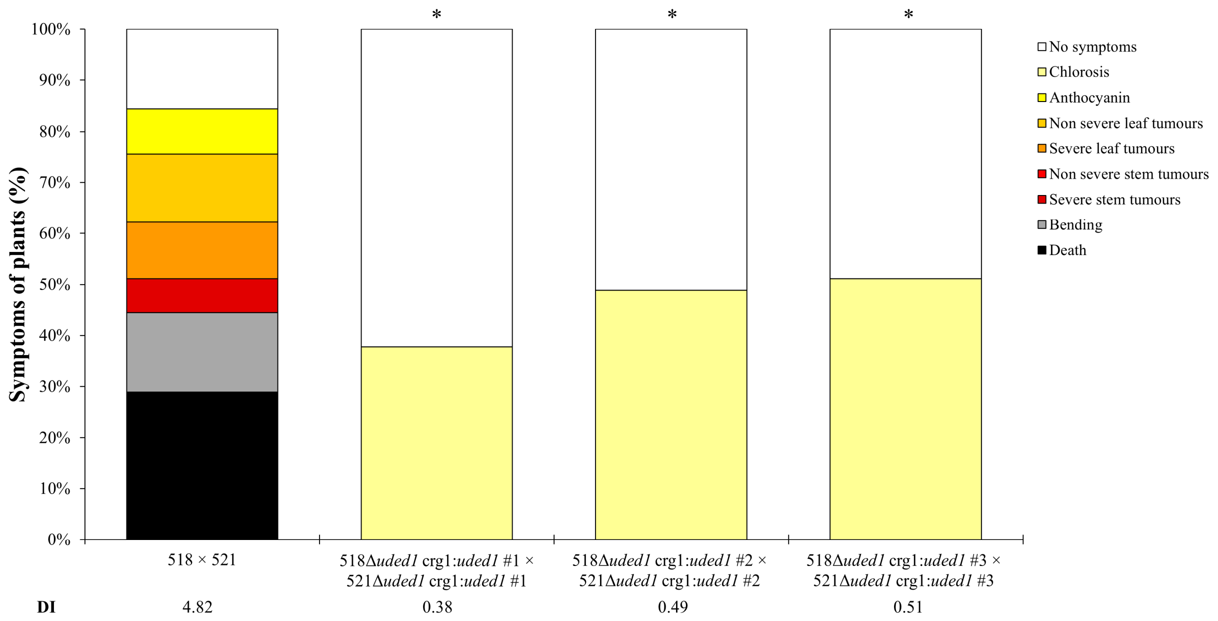

Figure S10. Pathogenesis assay of Δ*uded1* crg1:*uded1* mutant strains in maize seedlings. Evaluation of virulence of *Ustilago maydis* 518 × 521 (wildtype) and Δ*uded1* crg1:*uded1* × Δ*uded1* crg1:*uded1* (deletion) infections in *Z. mays*. Bars represent the percentage of plants displaying symptoms at 14 days post inoculation. Colours indicate the scored symptom ranging from mild (chlorosis) to severe symptoms (large stem tumours and plant death) as indicated in the legend. A total of 45 plants per cross were infected and the disease index (DI) is the calculated mean of the disease symptoms. Statistical differences were determined using Kruskal-Wallis test coupled with a Dunn multiple comparison ($p \leq 0.05$). Asterisks indicate a significant difference in virulence of deletion infections relative to wildtype infections (Mann-Whitney U-test, $p \leq 0.05$).

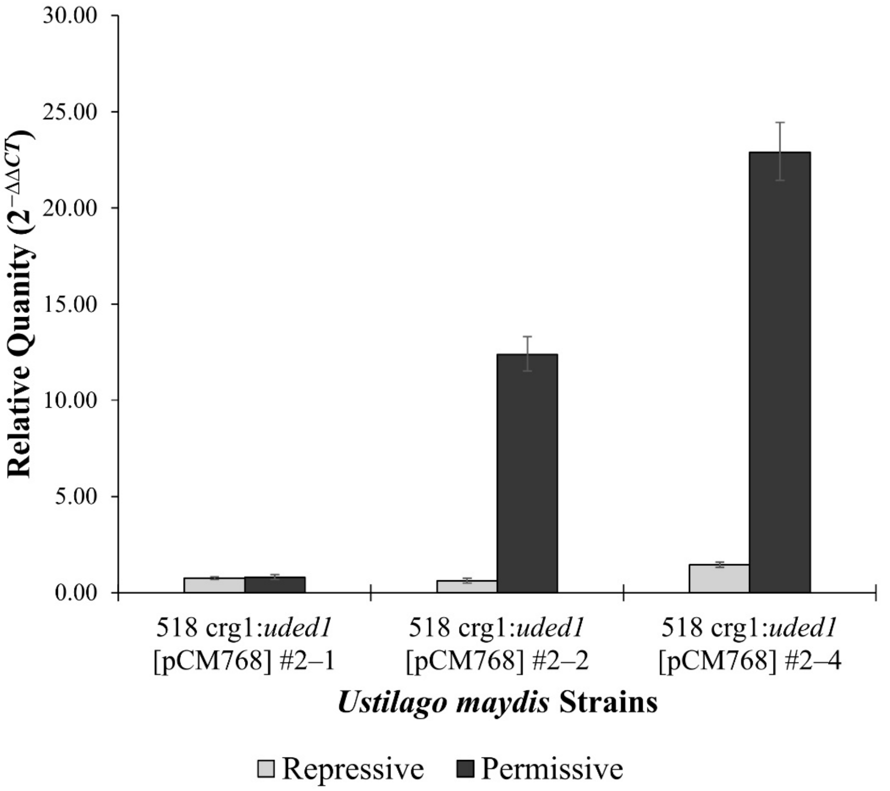

Figure S11. RT-qPCR analysis of *uded1* transcript levels in the 518 crg1:*uded1* [pCM768] control samples grown in repressive (YEPS) and permissive (YEPA) conditions. Relative quantities were calculated using the comparative C_T ($2^{-\Delta\Delta CT}$) method, *UMAG_00175* was the endogenous control, and the parent strain (518 crg1:*uded1*) grown in repressive conditions was set as the calibrator. Bars indicate the RQ minimum and RQ maximum values (95% confidence interval, n = 3).
